# Comparative Evaluation of a Multistrain Indirect ELISA Targeting Anti- p26 and gp45 Antibodies for EIAV Detection

**DOI:** 10.3390/pathogens14060575

**Published:** 2025-06-08

**Authors:** Angela Ostuni, Raffaele Frontoso, Maria Antonietta Crudele, Lorella Barca, Mario Amati, Raffaele Boni, Jolanda De Vendel, Paolo Raimondi, Alfonso Bavoso

**Affiliations:** 1 Department of Basic and Applied Sciences, University of Basilicata, Via dell’ Ateneo Lucano 10, 85100 Potenza, Italy; maria.crudele@unibas.it (M.A.C.); mario.amati@unibas.it (M.A.); raffaele.boni@unibas.it (R.B.); alfonso.bavoso@unibas.it (A.B.); 2 Istituto Zooprofilattico Sperimentale del Mezzogiorno, Via Salute, 2, 80055 Portici, Italy; lorella.barca@izsmportici.it; 3 OneHEco APS, 84047 Capaccio Paestum, Italy; jdevendel@tiscali.it (J.D.V.); paoloraimondi50@gmail.com (P.R.)

**Keywords:** EIAV, diagnostics, indirect ELISA, Bayesian latent class analysis, Cohen’s Kappa test

## Abstract

Equine Infectious Anemia Virus (EIAV), a lentivirus marked by considerable genetic variability, poses significant diagnostic challenges. Existing diagnostic tools encompass the Agar Gel Immunodiffusion Assay (AGID), enzyme-linked immunosorbent assay (ELISA), and Western blotting (WB). ELISA and AGID mainly utilize the p26 capsid protein, often sourced from the Wyoming reference strain. To broaden the range of viral proteins and strains employed in these immunoassays, we previously developed a novel p26/double-strain gp45 indirect ELISA. In this study, we evaluated the performance of this ELISA in comparison to two commercial EIAV ELISAs using Cohen’s Kappa test and Bayesian Latent Class Analysis (BLCA), a statistical method that estimates test performance without requiring a perfect reference standard. A comparison with the official classification of the sera by the Italian Veterinary Service was also performed. A total of 372 serum samples, including 96 that were positives by all three tests, were analyzed. Results from both Cohen’s Kappa test and BLCA, alongside comparison with official classifications, affirm the diagnostic reliability of the two commercial ELISAs and suggest that the novel ELISA, with its enhanced antigenic diversity, could offer an accurate and reliable diagnostic option for EIAV. This novel assay enhances existing commercial ELISAs and has the potential to strengthen routine diagnostic workflows.

## 1. Introduction

Equine Infectious Anemia Virus (EIAV) is a lentivirus belonging to the Retroviridae family that infects equines, including horses, mules, and donkeys. Similar to other lentiviruses like HIV and SRLV, EIAV establishes a lifelong infection characterized by phases of acute, chronic, and asymptomatic stages, where clinical manifestations range from febrile episodes to anemia and thrombocytopenia [1]. The virus is primarily transmitted by biting insects, such as Tabanidae, and can also spread via blood-contaminated instruments, presenting a serious concern for equine health worldwide. As a result, EIAV infections are subject to mandatory reporting to the World Organization for Animal Health (WOAH), with control measures typically involving isolation or euthanasia of infected animals [2].

EIAV’s high genetic variability, which categorizes it as a viral quasispecies, poses additional challenges to diagnostic accuracy and control. The global distribution of the virus encompasses a variety of genetic lineages that continually evolve through natural selection, leading to significant genetic variability [3,4,5,6]. Currently, diagnostic tools include the Agar Gel Immunodiffusion Assay (AGID), enzyme-linked immunosorbent assay (ELISA), and Western blotting (WB). AGID and ELISA primarily utilize the p26 capsid protein very often derived from the Wyoming reference strain [7,8,9,10]. While AGID remains a widely recognized standard, ELISAs have increasingly been used for initial screening due to higher sensitivity, followed by AGID and/or WB as confirmatory methods [9]. This three-tier approach has been successfully applied by the Italian Veterinary Service [10] since it has been shown that relying solely on agar gel immunodiffusion tests may not be sufficient for effective EIAV control [11,12,13]. Dependence on a single protein or strain can limit the sensitivity and specificity of these diagnostic tools, potentially failing to capture the full diversity of EIAV strains circulating in different regions.

Recent research has focused on expanding the range of viral proteins used in immunoassays. For instance, combining the gp45 transmembrane protein with p26 in ELISA tests has demonstrated enhanced sensitivity for antibody detection compared to p26-only assays [7,8]. In line with this approach, in a recent study [14], we utilized bioinformatics and molecular modeling to design suitable antigens for EIAV detection. The Blast analysis of the EIAV protein sequences deposited in GenBank showed a sequence variability of 18% concerning the p26 and 30% concerning the gp45 polypeptide, corresponding to a 145-residue exodomain of the transmembrane protein gp45. These values are very similar to the result obtained for the corresponding capsid and transmembrane proteins of SRLV [14]. Since the use of multistrain antigens of the capsid and transmembrane proteins is well established for the diagnosis of SRLV [15,16], we adopted a similar approach for EIAV. The resulting ELISA, which includes a double-strain gp45 exodomain of EIAV (https://www.sciencedirect.com/topics/pharmacology-toxicology-and-pharmaceutical-science/equine-infectious-anemia-virus, accessed on 27 March 2025) (residues 483–630 of strain “F3”, accession number AFW99173.1 and the corresponding residues of strain “POCONE-BRA1”, accession number QIC50013.1) and a p26 sequence of EIAV (https://www.sciencedirect.com/topics/agricultural-and-biological-sciences/equine-infectious-anemia-virus, accessed on 27 March 2025) (“native strain of Argentina”, accession number ABE03841.1), could demonstrate enhanced diagnostic sensitivity by capturing a broader range of viral variability, potentially addressing the limitations seen with a single protein, single-strain-based ELISA diagnostics. The new ELISA was not designed to replace the AGID as a confirmatory or official test, but to complement the existing ELISA as screening tool.

In the present study, we evaluated this novel p26/double-strain gp45 ELISA against two commercial EIAV ELISAs using Cohen’s Kappa test and Bayesian Latent Class Analysis (BLCA), a statistical method that estimates test performance without requiring a perfect reference test. Unlike traditional methods that rely on a single “gold standard”, BLCA incorporates information from multiple imperfect tests to estimate the sensitivity and specificity of each, accounting for potential dependencies between test results. This approach is particularly useful in veterinary virology, since a proper gold standard is often lacking, as in our case for EIAV detection. A comparison with the official classification of the sera by the Italian Veterinary Service was also performed.

## 2. Materials and Methods

### 2.1. Sample Collection

A total of 372 serum samples were used in this investigation, of which 272 samples were collected from animals in the Basilicata, Calabria and Campania Regions of Italy, while the remaining, of Italian and French origin, were provided from IZSLT (National Reference Center for EIA and WOAH Reference Laboratory for EIA) and were mostly positive. The test for EIAV is conducted by the Italian Veterinary Service following this procedure: an initial screening is performed using ELISA with one of the following commercial tests, IDvet (INNOVATIVE DIAGNOSTICS, Grabels, France) and IN3 Diagnostics (In3diagnostic s.r.l. Turin, Italy), or a test developed by the National Reference Center. If the ELISA result is positive, an AGID test is then performed; if the AGID is positive, the sample is classified as positive, if the AGID is negative, a Western blot is conducted to evaluate serum reactivity to three proteins—p26, gp45, and gp90. Finally, if the serum reacts to at least two of these proteins, the sample is considered positive [9].

All sampled animals were older than one year, as required by the surveillance program; no specific breed was selected in this study. All sampling procedures were conducted in agreement with European legislation, regarding the protection of animals used for scientific purposes (European Directive 2010/63) [17]. Veterinary officers from the Italian National Health System collected blood samples as part of the mandatory eradication and surveillance program for EIA, which requires periodic sampling of equines.

### 2.2. ELISA Assay Development and Testing

Details on the development of the in-house ELISA (ELISA 1) have been reported previously [14]. Briefly, this ELISA incorporates a recombinant double-strain gp45 transmembrane protein antigen alongside the p26 capsid protein to enhance sensitivity and capture the broad antigenic variability of EIAV. The GenBank database was exploited in a systematic approach to design these polypeptides. The main bioinformatic tools used were clustering, molecular modeling, epitope predictions and aggregative/solubility predictions. The gp45 antigen was developed by fusing two representative gp45 variants (residues 483–630 of strain “F3”, accession number AFW99173.1 from Ireland and the corresponding residues of strain “POCONE-BRA1”, accession number QIC50013.1 from Brasil) with maltose-binding protein at the N-terminus to enhance solubility. The p26 was selected for its broad reactivity across EIAV strains (“native strain of Argentina”, accession number ABE03841.1). Both proteins were expressed in *Escherichia coli* starting from synthetic genes and purified by affinity chromatography (HisTrap HP column, Merck KGaA, Darmstadt, Germany). For the assay, microtiter plates were coated overnight at 4 °C with a mixture of gp45 and p26 antigens in a 5:1 mass ratio (0.5 μg of gp45 antigen and 0.1 μg of p26 per well). Plates were then blocked with 2% ovalbumin (Merck KGaA, Darmstadt, Germany) in phosphate-buffered saline (PBS) to minimize non-specific binding. Serum samples were diluted 1:200 in PBS containing 0.05% Tween-20 (Società Italiana Chimici Life Science, Rome, Italy) (PBS-T) and applied to the plates in duplicate, followed by a one-hour incubation at room temperature. Plates were washed with PBS-T, and Protein G conjugated to horseradish peroxidase (HRP) (Merck KGaA, Darmstadt, Germany), diluted 1:60,000 in PBS-T, was added as the secondary antibody. The colorimetric reaction was developed using tetramethylbenzidine (TMB) (Merck KGaA, Darmstadt, Germany) substrate, stopped after 15 min with 1 N sulfuric acid, and absorbance was measured at 450 nm using a microplate reader (Multiskan TM GO Microplate Spectrophotometer, Thermo Scientific, Waltham, MA, USA). Per cent reactivity was calculated using the formula S/P% = [(OD_sample_ − OD_negative control_)/(OD_positive control_ − OD_negative control_)] × 100. As already reported, a receiver operating characteristic (ROC) analysis of data established the threshold (50%) for positive/negative classification; therefore, samples with S/P% ≥ 50% were considered positive.

### 2.3. Commercial ELISA

Two commercial EIAV ELISAs, ELISA 2 (IDvet EIA, IDvet, Grabels, France) [7] and ELISA 3 (Ingezim DR EIA, Ingenasa, Madrid, Spain) [8,18], were used for comparison with ELISA 1 (the in house developed test). Both commercial double-recognition (DR) ELISAs detect antibodies against the EIAV p26 protein, likely derived from the Wyoming strain. In both assays, the p26 antigen is first coated onto the plate to capture EIAV-specific antibodies in the serum. A second p26 antigen, conjugated to horseradish peroxidase (HRP), is then added to bind to the captured antibodies, effectively “sandwiching” the antibodies between two layers of p26. This setup ensures high specificity in detecting EIAV antibodies. Testing was performed according to the manufacturers’ instructions. In ELISA 2, S/P% = [(OD_sample_ − OD_negative control_)/(OD_positive control_ − OD_negative control_)] × 100 is used. According to the instructions, samples are classified as follows: S/P% ≤ 50% negative, 50 < S/P% < 60 dubious and S/P% ≥ 60% positive. In this analysis, we considered samples with S/P% > 50% as positive. In ELISA 3, the value OD_sample_/OD_positive control_ (S/P) is calculated; samples are considered positive if S/P > 0.3.

### 2.4. Statistical Analysis

The agreement between the results of the in-house test as compared to the two commercial ELISA was assessed using Cohen’s Kappa analysis with the aid of an online calculator [19]. The agreement results were classified into six categories based on kappa values (0–1): slight (0–0.20), fair (0.21–0.40), moderate (0.41–0.60), substantial (0.61–0.80), and almost perfect (0.80–1.0) agreements [20].

The performance of the three ELISA tests was also evaluated using Bayesian Latent Class Analysis (BLCA) where all samples were assumed to represent a single population. The model estimates the diagnostic sensitivity (Dse) and diagnostic specificity (Dsp) for each of the three tests as well as the true prevalence of the population. The calculation was performed with a web-based software available online [21] (here referred as BLCA_A) using the 3-tests-in-1 population model (Advanced Interface); it is an application of WinBUGS statistical software for Bayesian analysis (Version 1.4.3) [22]. Default input parameters were used. To ensure objectivity in parameter estimation, Jeffreys prior, a non-informative prior distribution represented as a Beta (0.5, 0.5) distribution for Dse, Dsp and prevalence, was used; it allows for neutral assumptions about these parameters before observing the data and ensures that the data drive posterior estimates. In total, 25,000 iterations were run, with a burn-in of 5000, and 1/10 thinning across 2 chains, giving 2000 posterior samples. The median and 95% posterior credible intervals (PCIs) for the estimated parameters from the posterior samples were calculated. Positive predictive values (PPVs) and negative predictive values (NPVs) for each test were also calculated. The convergence of the models was assessed using visual inspection of the Markov chain Monte Carlo (MCMC) trace plots.

In addition, a second software (referred here as BLCA_B) obtained by a minimal modification of a published R script was also employed [23]; it is an implementation of JAGS, a program for Bayesian inference using MCMC methods. A Jeffreys distribution was assumed for Dse and Dsp for each test and prevalence. The uninformative, uniform distribution Beta (1,1) was also tested, yielding less satisfactory results. Two simulations were performed; in one case, the data were considered a single population, and in the second case, the data were divided into three almost equivalent populations. In the first calculation, 405,000 iterations were conducted, with a burn-in of 5000 and a thinning of 1/20 across two chains. In the second calculation, only the number of iterations (205,000) was modified. Convergence of the models was assessed again through visual inspection of the MCMC trace plots and by calculating the potential scale reduction factor (PSRF) of the Gelman–Rubin statistic, which was less than 1.05 for all parameters in both simulations. The median and 95% PCI were calculated to summarize the uncertainty around sensitivity, specificity, and prevalence estimates for each test. PPV and NPV for each test were also obtained from the calculations.

Dse, Dsp, PPV, NPV and their respective CI were calculated for the ELISAs with respect to the sera official classification by the Italian Veterinary Service assumed as the gold standard. A custom R script was applied and Wilson’s score method was used to compute 95% confidence intervals for each metric.

## 3. Results

A total of 372 serum samples, including 96 samples that were positive for all three tests, were analyzed. A summary of the ELISA results is presented in Table 1. The calculated Cohen’s Kappa values, along with the respective confusion matrices, are displayed in Table 2 for comparison of ELISA 1 with both commercial tests and with the Official Classification data. The agreement between ELISA 1 and ELISA 2 was 99.2%, with a Cohen’s Kappa of 0.980 (95% CI: 0.957–1.000). The agreement between ELISA 1 and ELISA 3 was 98.7%, with a Cohen’s Kappa of 0.965 (95% CI: 0.935–0.996). Finally, the agreement between ELISA 1 and the Official Classification was 99.2%, with a Cohen’s Kappa of 0.980 (95% CI: 0.957–1.000). The results indicate almost perfect agreements, according to the aforementioned classification. Detailed values for Dse, Dsp, PPV and NPV calculated with both BLCA_A and BLCA_B software in the single population assumption, together with the results obtained with respect to the Official Classification, are reported in Table 3.

## 4. Discussion

This study assessed the effectiveness of an in-house indirect ELISA designed for detecting Equine Infectious Anemia Virus (EIAV) antibodies, reaching sensitivity and specificity rates exceeding approximately 98%. This high performance is particularly significant given EIAV’s genetic variability, which comprises multiple strains that can elicit diverse immune responses in affected equine populations. Compared to other indirect ELISAs that typically rely on a single p26 antigen, our assay combines p26 with a double-strain gp45, broadening epitope coverage and potentially improving sensitivity across diverse EIAV variants. Diagnostic applications of the gp45 peptides have already been suggested in the literature [24,25,26]. Unlike DR ELISAs, which use a dual-p26 sandwich format and are limited to a single antigen, the indirect format allows more flexibility in incorporating multiple antigens. However, indirect ELISAs may be more susceptible to background noise and depend on high-quality secondary reagents for optimal performance. Thus, while the format is standard, our antigenic design enhances the utility of the indirect ELISA for use in variable field conditions.

To rigorously compare the performance of our indirect ELISA with two Double Recognition ELISAs (DR ELISAs), we utilized Bayesian Latent Class Analysis on a dataset comprising 372 samples. This statistical approach is highly recommended by WOAH when a gold standard is unavailable [27,28]. It provides a robust framework for evaluating diagnostic tests, allowing for the integration of prior knowledge and the calculation of posterior probabilities. Two BLCA softwares were used based on WinBUGS and JAGS, respectively, which differ in their underlying architecture and computational processes. The results of our ELISA were also compared with those from the Official Classification of the sera. Very similar results were obtained for the same test in all the evaluations.

Results concerning the prevalence are evidently displayed due to the artificial nature of the samples’ population. Calculations with BLCA_B were performed using both a single-population and a three-population model. The rationale for adopting a three-population model was to assess whether incorporating additional variables could enhance the fit of the Bayesian model to the data. This is a common consideration in BLCA, where the results are sensitive to the model structure and the number of parameters being estimated. The similarity in results across models supports the robustness of the single-population approach. In fact, results obtained with BLCA_B in the tree population assumption are very similar to those from the single population calculation (Supplementary Table S1); this is likely due to the choice of dividing the dataset into three almost equivalent populations. However, it is worth noting that convergence was reached with fewer iterations in the case of three populations. The sensitivity, specificity, and predictive values of each ELISA test were reported along with their 95% PCIs, reflecting the precision of the estimates. When comparing the BLCA results, the two models (BLCA_A and BLCA_B) displayed slight differences in their estimates for sensitivity, specificity, PPV, and NPV. Such differences may arise from the application of varying model assumptions, as is typically the case in Bayesian analysis. All three ELISAs demonstrate high sensitivity (≥95%) and specificity (≥98%), with slightly differing predictive values. ELISA 1 (the novel assay) exhibited very good diagnostic sensitivity (BLCA_A: 98.8%, BLCA_B: 98.0%) and diagnostic specificity (BLCA_A: 99.9%, BLCA_B: 99.6%), along with high PPV and NPV, reinforcing its diagnostic reliability.

In comparing the tests, ELISA 1 demonstrated slightly lower sensitivity compared to ELISA 2 but exhibited similar specificity. ELISA 3 demonstrated a minimal decrease in performance in Dse in relation to both ELISA 1 and ELISA 2. When considering the results from the comparison with the Official Classification, no significant differences were observed with respect to the BLCA of the three ELISAs. It is worth noting that ELISA 2 was in perfect agreement with the Official Classification. However, we should consider that ELISA 2 is one of the three ELISAs currently approved and used as a screening tool by some laboratories of the Italian Veterinary Service and this could introduce certain level of bias in the data used as the gold standard. These results confirmed the validity of both commercial ELISA tests for diagnostic screening, in agreement with their reported performances [7,8,18].

The results from this analysis revealed that our indirect ELISA demonstrates sensitivity and specificity levels comparable to those of the commercial DR ELISAs. The results suggested that the novel ELISA, with its enhanced antigenic diversity, could provide a valid diagnostic option for EIAV, complementing existing commercial ELISAs and strengthening routine diagnostics. Overall, these data support the reliability of the p26/double-strain gp45 ELISA for EIAV detection.

The DR ELISAs, employing a single antigen in a double recognition format, increases the likelihood of detecting even low concentrations of antibodies, enhancing their overall diagnostic sensitivity. The DR format could also enhance its specificity. However, while the low background noise from this method is beneficial, it may also inadvertently restrict the range of antibody recognition to only those that target the single antigen used.

In contrast, the in-house indirect ELISA is designed to address potential limitations associated with antigen variability through its dual-antigen, multi-strain design. The indirect ELISA’s ability to handle antigen variability makes it a valuable tool for routine diagnostic workflows, especially in areas with diverse EIAV strains. This adaptability could support more effective control and eradication programs by reducing the risk of false negatives associated with strain-specific limitations.

However, it is worth noting that AGID undoubtedly remains the gold standard for confirmation purposes due to its high specificity as compared with ELISA [29,30,31].

## 5. Conclusions

Although the studied assay uses a standard indirect ELISA format commonly found in EIAV diagnostics, its distinguishing feature lies in using multi-strain antigens—combining p26 with double-strain gp45—with the aim of improving sensitivity across diverse EIAV variants. This contrasts with other ELISAs that generally rely on a single antigen. The indirect format also offers flexibility in antigen design with respect to DR ELISA, though it requires high-quality reagents to minimize background noise. The use of synthetic genes selected through bioinformatics enables easy future adaptation to local EIAV strains. Additionally, the study employed a rigorous statistical framework—Cohen’s Kappa and Bayesian Latent Class Analysis—to ensure reliable comparison in the absence of a gold standard, with consistent results across both WinBUGS and JAGS implementations supporting the assays’ diagnostic accuracy.

## Figures and Tables

**Table 1:** Summary of the ELISA results.

ELISA 1	ELISA 2	ELISA 3	Frequency Observed
Positive	Positive	Positive	96
Positive	Positive	Negative	3
Positive	Negative	Positive	0
Negative	Positive	Positive	1
Positive	Negative	Negative	0
Negative	Positive	Negative	2
Negative	Negative	Positive	1
Negative	Negative	Negative	269

ELISA 1 = in-house ELISA; ELISA 2 = IDvet EIA; ELISA 3 = Ingezim DR EIA. All three tests were performed on 372 serum samples.

**Table 2:** Calculated Cohen’s Kappa values from comparing in-house ELISA (ELISA 1) with both the commercial tests, ELISA 2 = IDvet EIA and ELISA 3 = Ingezim DR EIA, and the Official Classification.

ELISA 1	Negative	Positive	Total	Observed Agreements (%)	Agreement Expected by Chance (%)	Kappa Cohen (CI)	Kappa Cohen (SE)
ELISA 2: **Negative**	270	0	270				
ELISA 2: **Positive**	3	99	102				
**Total**	273	99	372				
				369 (99.19)	225.3 (60.56)	0.980 (0.957–1.000)	0.012
ELISA 3: **Negative**	271	3	274				
ELISA 3: **Positive**	2	96	98				
**Total**	273	99	372				
				367 (98.66)	227.2 (61.06)	0.965 (0.935–0.996)	0.015
Official Classification: **Negative**	270	0	270				
Official Classification: **Positive**	3	99	102				
**Total**	273	99	372				
				369 (99.19)	225.3 (60.56)	0.980 (0.957–1.000)	0.012

Confusion matrix and Cohen’s Kappa values for comparison of ELISA1 with ELISA 2, ELISA 3, and Official Classification. CI: 95% confidence interval; SE: standard error.

**Table 3:** Diagnostic sensitivities and specificities and positive and negative predictive values (PPVs and NPVs) estimated by using Bayesian Latent Class model and two softwares BLCA_A and BLCA_B and calculated with respect to the Official Classification of the Italian Veterinary Service.

Parameters	BLCA_A (%)	BLCB_B (%)	Official Classification
**ELISA 1**			
Sensitivity	98.8 (95.3–99.9)	98.0 (93.0–100)	97.1 (91.7–99.0)
Specificity	99.9 (99.1–100)	99.6 (98.0–100)	100 (98.6–100)
PPV	99.8 (97.5–100)	99.0 (98.1–99.4)	100 (96.3–100)
NPV	99.6 (98.2–100)	99.3 (98.5–99.6)	98.9 (96.8–99.6)
**ELISA 2**			
Sensitivity	99.8 (97.5–100)	98.9 (94.6–100)	100 (96.4–100)
Specificity	99.9 (99.1–100)	98.9 (97.2–100)	100 (98.6–100)
PPV	99.8 (97.6–100)	97.2 (96.0–98.1)	100 (96.4–100)
NPV	99.9 (99.1–100)	99.6 (98.9–99.8)	100 (98.6–100)
**ELISA 3**			
Sensitivity	96.8 (92.5–99.1)	95.7 (90.8–100)	95.1 (89.0–97.9)
Specificity	99.6 (98.2–100)	99.2 (97.6–100)	99.6 (97.9–99.9)
PPV	98.8 (95.2–99.9)	97.9 (96.8–98.6)	99.0 (94.4–99.8)
NPV	98.8 (97.2–99.7)	98.4 (97.4–99.0)	98.2 (95.8–99.2)

Positive and negative predictive values, PPVs and NPVs, respectively. The 95% confidence interval is shown in brackets.

## Data Availability

The original contributions presented in this study are included in the article. Further inquiries can be directed to the corresponding authors.

## Supplementary Materials

The following supporting information can be downloaded at: https://www.mdpi.com/article/10.3390/pathogens14060575/s1, Table S1: Diagnostic sensitivities, specificities, positive and negative predictive values (PPVs and NPVs) estimated by using Bayesian Latent Class model BLCA_B 3 populations.
