# Supplementary material for: Comparative Evaluation of a Multistrain Indirect ELISA Targeting Anti- p26 and gp45 Antibodies for EIAV Detection
Source: Pathogens. 2025 Jun 8;14(6):575. doi: 10.3390/pathogens14060575 (PMC12196156; doi:10.3390/pathogens14060575)
Supplement: Supplementary file 1 [file pathogens-14-00575-s001.zip › pathogens-3583744-supplementary.pdf]

Supplementary Table S1. Diagnostic sensitivities, specificities, positive and negative predictive values (PPVs and NPVs) estimated by using Bayesian Latent Class model BLCA_B 3 populations.

Test	Sensitivity (%)	Specificity (%)	PPV	NPV
**ELISA 1**	98.2 (93.5 – 100.0)	99.6 (98.2 – 100.0)	99.0 (98.1 – 99.4)	99.3 (98.6 – 99.7)
**ELISA 2**	98.6 (94.9 – 100.0)	98.5 (96.8 – 100.0)	96.0 (94.6 – 97.0)	99.5 (98.8 – 99.8)
**ELISA 3**	97.0 (92.4 – 100.0)	99.3 (97.8 – 100.0)	98.2 (97.1 – 98.8)	98.9 (98.1 – 99.4)

The 95% confidence interval is shown in brackets.
